# Peppermint oil in menstrual disorders and menopausal symptoms: a research progress review

**DOI:** 10.3389/fendo.2026.1880212

**Published:** 2026-07-22

**Authors:** Linfeng Mo, Shuya Ye, Yan Qin, Huihuang Shen, Huiyi He, Wenyan Hong, Hongmei Wu

**Affiliations:** 1 Department of Health Management, Guangzhou Huashang Vocational College, Guangzhou, China; 2 Department of Hospital Management, Hangzhou Children’s Hospital, Hangzhou, China; 3 Department of Epidemiology and Health Statistics, Guilin Medical University, Guilin, China

**Keywords:** dysmenorrhea, menopausal symptoms, menstrual disorders, peppermint oil, precision medicine

## Abstract

Globally, approximately 80–95% of women of reproductive age are affected by premenstrual syndrome and menstrual-related symptoms, which significantly impair their quality of life. Although non-steroidal anti-inflammatory drugs and hormonal therapies are considered first-line treatment options, they are limited by adverse effects and restrictions on applicable populations. Peppermint oil is a natural plant extract. Its primary bioactive component, menthol, exhibits significant antioxidant, anti-inflammatory, and smooth muscle relaxant pharmacological activities. It has shown preliminary application potential in the management of menstrual disorders and menopausal symptoms, yet it is still in the early stage of research. This review synthesizes current research progress on peppermint oil in the management of menstrual disorders and menopausal symptoms, with a focus on its pharmacological basis, clinical application evidence, and existing controversies and research gaps. Available evidence indicates that peppermint oil exerts preliminary efficacy in alleviating primary dysmenorrhea and improving genitourinary symptoms of menopause. These effects are mediated by multi-target mechanisms, including ion channel modulation, inhibition of inflammatory pathways, and regulation of the neuroendocrine network. However, current research faces key bottlenecks, including fragmented clinical evidence, unclear mechanisms of action, lack of product quality standardization, and insufficient long-term safety data. To address these challenges, future efforts should prioritize large-scale, standardized randomized controlled trials. In parallel, future research may also integrate multi-omics technologies to elucidate its molecular mechanisms and develop novel drug delivery systems to enhance bioavailability. We hypothesize that this pathway holds promise for moving peppermint oil from traditional experience toward evidence-based medicine, though the precision medicine framework remains an exploratory direction given the current state of evidence.

## Graphical Abstract

**Figure:**
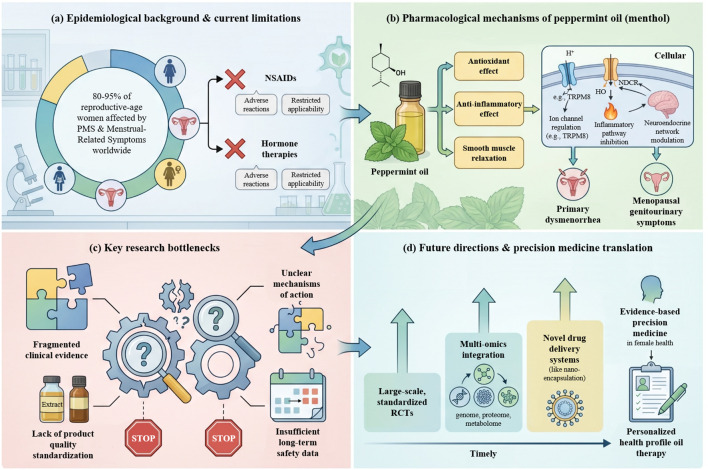

## 1 Introduction

Menstrual disorders represent one of the most common health issues among women of reproductive age, primarily encompassing primary and secondary dysmenorrhea. Primary dysmenorrhea is defined as menstrual pain occurring in the absence of any underlying pelvic pathology. Its core mechanism involves excessive secretion of endometrial prostaglandins, particularly prostaglandin F2α (PGF2α). This prostaglandin induces excessive and uncoordinated contractions of the uterine smooth muscle, subsequently leading to ischemic pain (1–3). This condition often coexists with abnormal bleeding patterns, such as heavy menstrual bleeding and prolonged menstruation, suggesting possible common disturbances in endometrial or vascular regulation (4). Furthermore, psychological factors, including anxiety and depression, can significantly increase the risk of dysmenorrhea and exacerbate its severity. These findings underscore the important role of psycho-physiological interactions in the pathogenesis of this condition (5). Secondary dysmenorrhea is typically caused by organic pathologies such as endometriosis and adenomyosis (1, 6), necessitating a differential diagnosis from primary dysmenorrhea in clinical practice.

Epidemiological data show that approximately 80–95% of women of reproductive age experience menstrual-related symptoms, resulting in a significantly diminished quality of life (7). Among specific populations, such as healthcare workers and athletes, the risk of menstrual disorders is particularly pronounced (3, 8, 9). Currently, non-steroidal anti-inflammatory drugs (NSAIDs) and oral contraceptives remain the first-line agents for managing menstrual disorders, including dysmenorrhea (10). However, NSAIDs are associated with adverse effects, including gastrointestinal and cardiovascular events (11, 12), while oral contraceptives exhibit interindividual variability in efficacy and pose restrictions on applicable populations (3, 13). For secondary dysmenorrhea, hormonal therapies such as the etonogestrel implant have demonstrated certain therapeutic benefits (6); nevertheless, their safety profile requires further validation. Hormone replacement therapy (HRT) for menopausal syndrome also faces long-standing controversies regarding long-term safety. Moreover, the existing studies suffer from methodological flaws such as small sample sizes and inadequate implementation of blinding, which reduces the reliability and generalizability of the evidence (6, 14). Against this background, the pursuit of safe and effective complementary and alternative therapies has become an important direction.

Complementary and alternative medicine, represented by phytotherapy and aromatherapy, is increasingly being applied in the field of gynecology. For example, blood-activating and stasis-resolving herbs and their essential oils have become common alternative options for patients managing dysmenorrhea, owing to their effects on regulating uterine contractions and inhibiting inflammatory pathways (15). Modern studies have confirmed that essential oils such as those from Citrus species and lavender can ameliorate premenstrual symptoms and improve menopausal sleep quality (7, 16–19). Peppermint oil is a natural plant extract containing terpenoids, including menthol and menthone, as its core bioactive components. These compounds exhibit significant antioxidant, anti-inflammatory, and smooth muscle relaxant properties (20), demonstrating considerable potential in alleviating menstrual disorders and menopausal symptoms. However, its efficacy and safety require systematic evaluation. Basic research has provided preliminary evidence for the modulatory effects of peppermint oil on the reproductive system (21). At the cellular level, menthol has been shown to inhibit prostaglandin synthesis in endometrial cells and exert anti-inflammatory effects through modulation of the MAPK and PI3K-Akt signaling pathways (22). In lipopolysaccharide-stimulated macrophage models, the inhibitory effect of menthol on NF-κB activation has been verified, further supporting its potential to modulate inflammatory signaling pathways (23). Animal studies have provided complementary evidence, with peppermint essential oil also demonstrating anti-inflammatory effects in a 2, 4-dinitrochlorobenzene-induced mouse model of allergic dermatitis (24). Collectively, these findings suggest, at both cellular and animal levels, that peppermint oil possesses anti-inflammatory activity and the ability to modulate uterine contractility. However, the evidence remains fragmented and largely derived from non-reproductive-specific models, and the long-term effects on reproductive tissues remain unclear. However, existing studies have largely focused on the analgesic effects of peppermint oil. There remains a marked lack of systematic investigation into its regulatory effects on the menstrual cycle and menopausal symptoms, as well as its underlying molecular mechanisms (14, 25). Furthermore, clinical trials are hampered by heterogeneity issues, including small sample sizes, inconsistent intervention protocols, and substantial variability in outcome measures. These issues severely limit evidence synthesis and clinical translation (7, 14).

Therefore, to ensure a comprehensive overview, we conducted a literature search in PubMed, Scopus, Web of Science, Science Direct, Google Scholar, and CNKI databases from inception to April 2026 using keywords including “peppermint oil, ” “menthol, ” “*Mentha piperita*, ” “dysmenorrhea, ” “menstrual disorders, ” “premenstrual syndrome, ” “menopause, ” “menopausal symptoms, ” “hot flashes, ” and “female reproductive health.” Only peer-reviewed articles in English were included, and reference lists were manually screened. This review aims to systematically synthesize the available evidence on peppermint oil in the context of menstrual disorders and menopausal symptoms, integrate its pharmacological mechanisms with current clinical research, and critically analyze the key challenges facing the field. Through this integrated analysis, we hope to provide a theoretical basis and research framework to inform future directions.

## 2 Pharmacological basis of peppermint oil

The biological activity of peppermint oil derives from its complex chemical composition and the interactions among its constituents. A thorough understanding of its chemical profile and pharmacological mechanisms is fundamental to elucidating its therapeutic value in female reproductive health.

### 2.1 Complex chemical composition as the material basis for multiple pharmacological activities

The core biological activity of peppermint oil is primarily attributed to its monoterpenoid constituents, among which menthol is the most abundant component (26, 27). Modern analytical techniques, such as chiral column chromatography, have enabled the separation of the eight stereoisomers of menthol. Studies have revealed that only specific configurations, such as (1R, 3R, 4S)-(-)-menthol and (1R, 3S, 4S)-(+)-neomenthol, are predominant in natural peppermint (28). This finding provides a molecular-level explanation for the variability in biological activity observed among peppermint oils from different sources. In addition to menthol, other key components include menthone and limonene. Notably, the essential oil composition differs significantly among peppermint varieties (e.g., *Mentha* × *piperita* and *Mentha spicata*), directly leading to heterogeneity in their antioxidant, anti-inflammatory, and antispasmodic activities (29–31). The Flavor and Extract Manufacturers Association of the United States (FEMA) has recognized several peppermint oils, including peppermint oil (*Mentha* × *piperita*), as Generally Recognized as Safe (GRAS) substances (30). Furthermore, extraction techniques significantly influence component composition and yield. Novel biomolecular heating distillation technology has been shown to substantially increase oil yield and menthol content compared with conventional water distillation (32), providing a technical direction for standardized production.

### 2.2 Antioxidant and Anti-inflammatory activities: targeting the MAPK/PI3K-Akt signaling axis

**Figure 1:**
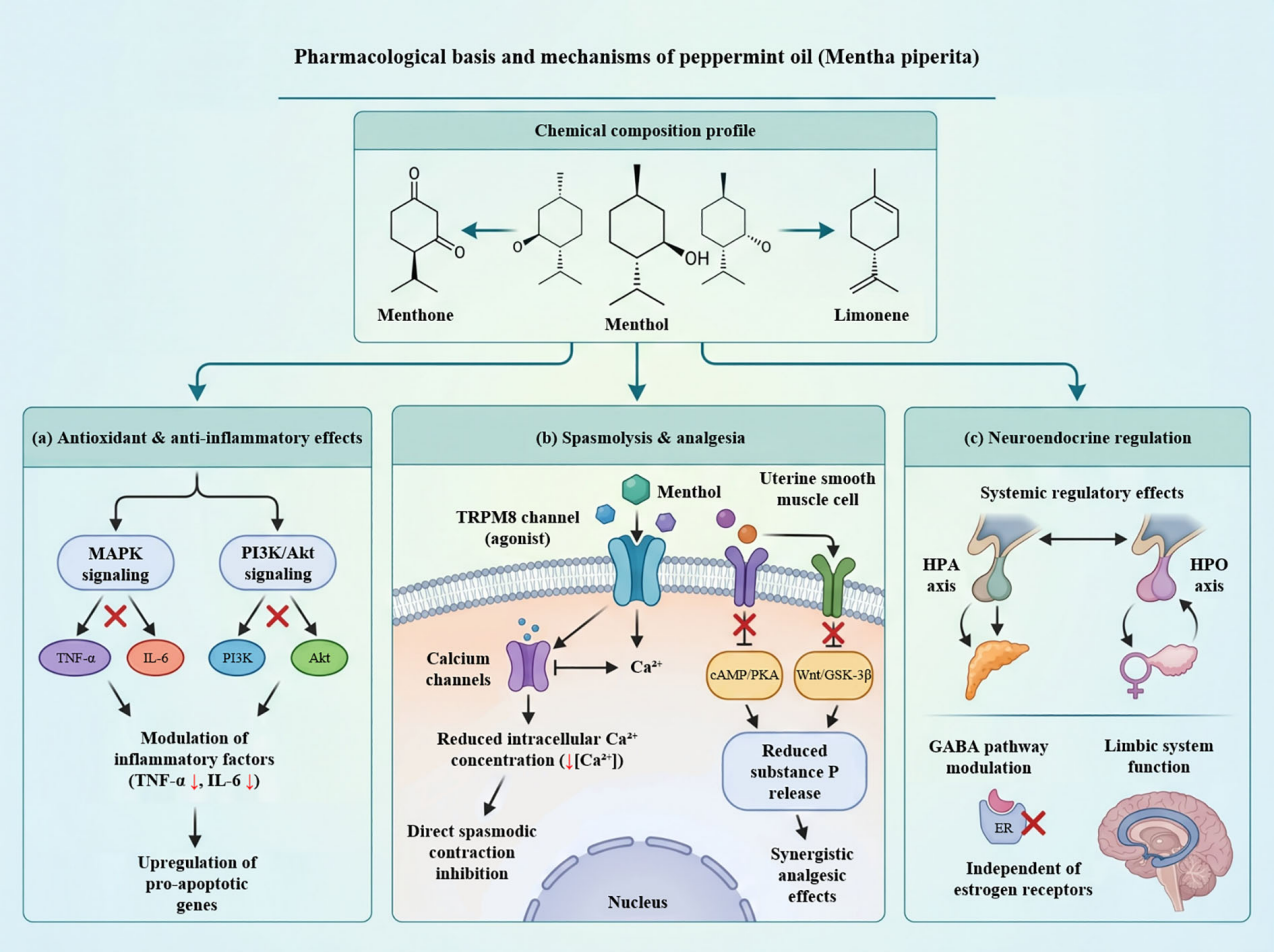
Multi-target mechanisms of peppermint oil in dysmenorrhea and menopausal symptoms. **(a)** Antioxidant and anti‑inflammatory effects via modulation of MAPK and PI3K/Akt signaling pathways, regulation of inflammatory cytokines (TNF‑α, IL‑6), and upregulation of pro‑apoptotic genes. **(b)** Spasmolysis and analgesia through TRPM8 channel agonism, calcium channel modulation, and inhibition of cAMP/PKA and Wnt/GSK‑3β pathways to reduce uterine smooth muscle contraction and substance P release. **(c)** Neuroendocrine regulation involving the HPA and HPO axes, GABA pathway modulation, and limbic system function, with effects likely independent of estrogen receptor activation.

The antioxidant and anti-inflammatory activities of peppermint oil constitute the molecular basis for its intervention in inflammation-related reproductive disorders, such as dysmenorrhea and endometriosis. Studies have shown that esterification or amination modification of menthol can enhance its biological activity (26). At the cellular level, peppermint oil exerts its effects by modulating key signaling pathways, including MAPK and PI3K-Akt. This leads to the induction of apoptosis, inhibition of invasion and migration, cell cycle arrest, and upregulation of pro-apoptotic genes such as Bax and p53 (**Figure 1a**) (22). Concurrently, peppermint oil modulates the levels of inflammatory cytokines, including tumor necrosis factor (TNF) and interleukins (e.g., IL-6, IL-8) (22). Its low toxicity, high efficacy, and well-defined antioxidant potential collectively form the molecular foundation for its anti-inflammatory effects, providing a theoretical basis for its intervention in inflammation-related reproductive disorders. Beyond inflammatory diseases of the reproductive system, emerging studies have suggested therapeutic potential for peppermint oil and its constituent menthol in other inflammatory conditions. For instance, randomized controlled trials (RCTs) have reported that topical application of peppermint oil reduces pain and improves joint function in patients with knee osteoarthritis (33). In animal models of rheumatoid arthritis, menthol administration alleviated joint swelling and downregulated pro-inflammatory cytokine expression (34). Although these studies are not directly related to reproductive health, they provide supportive evidence for the broader anti−inflammatory properties of peppermint oil, suggesting that its mechanisms (e.g., TRPM8 activation and NF−κB inhibition) may have commonality in different inflammatory contexts. Future research could further explore whether these anti−inflammatory effects are applicable to gynaecological inflammatory conditions, such as endometriosis or pelvic inflammatory disease.

### 2.3 Antispasmodic and analgesic effects: TRPM8 channel-mediated uterine smooth muscle relaxation

The efficacy of peppermint oil in alleviating dysmenorrhea is primarily attributed to its direct peripheral smooth muscle relaxant effects and indirect central/peripheral analgesic effects. Its main component, menthol, possesses well-defined antispasmodic and smooth muscle relaxant properties (35), particularly exhibiting specific regulatory capacity on uterine smooth muscle. On one hand, menthol acts as an agonist of the transient receptor potential melastatin 8 (TRPM8) channel. By modulating calcium channels, it reduces intracellular calcium concentrations in uterine smooth muscle cells, thereby directly inhibiting spasmodic contractions (35, 36). On the other hand, menthol may reduce the release of the pain neurotransmitter substance P by inhibiting PKAca phosphorylation in the cAMP signaling pathway and GSK-3β in the Wnt/β-catenin pathway (**Figure 1b**) (37). This produces synergistic peripheral and central analgesic effects. Notably, the TRP channel family (e.g., TRPM8 and TRPA1) plays a key role in these processes (38). Furthermore, thymol, a related component of peppermint oil, can also reduce uterine contraction intensity (39), whereas turmeric rhizome essential oil exerts antispasmodic effects by activating L-type calcium channels (40). Although these findings are not direct evidence for peppermint oil, they suggest that different essential oil components may synergistically enhance spasmolytic effects through complementary mechanisms. Animal studies further indicate that peppermint oil improves ovarian function in rat models of polycystic ovary syndrome (PCOS). In a PCOS−induced rat model, administration of peppermint oil reduced body weight, testosterone levels, ovarian cysts, and the number of follicles without ovum, while increasing Graafian follicles. This suggests that peppermint oil may improve ovarian function through anti−androgenic effects (41). However, it should be noted that this evidence comes from a single small−sample animal study, and its relevance to human PCOS, a heterogeneous disorder with complex metabolic and endocrine disturbances, remains to be verified. Future studies employing preclinical models with dose−response designs are warranted.

### 2.4 Neuroendocrine regulation: systemic effects mediated by HPO-HPA axis interaction

Beyond its local effects, peppermint oil may exert systemic influences by modulating the neuroendocrine network. If validated, this hypothesis could represent a potential advantage that distinguishes it from conventional NSAIDs. Animal studies have demonstrated that menthol can regulate the function of the hypothalamic-pituitary-ovarian (HPO) axis (42), and that the HPO axis exhibits crosstalk with the hypothalamic-pituitary-adrenal (HPA) axis (**Figure 1c**) (43–45). These findings suggest that peppermint oil may relieve stress-related menstrual disorders and menopausal mood disturbances through these two neuroendocrine networks. Regarding the management of menopausal psychological symptoms, indirect evidence supports that aromatherapy inhalation may influence neuroendocrine function by acting on the limbic system. For example, lavender essential oil has been shown to improve sleep and life satisfaction in menopausal women (18, 46), while fir essential oil helps promote relaxation (47). Although the above studies were not directly conducted on peppermint oil, they provide a reference for understanding how essential oils may influence menopausal psychological status through neuroendocrine pathways. In studies specifically on peppermint oil, menthol possesses well-defined neuroreceptor binding activity, and experimental evidence suggests it may influence anxiety-related behaviors by modulating pathways such as γ-aminobutyric acid (GABA) (48, 49). However, a critical question remains unresolved: does peppermint oil directly interact with estrogen receptors? Current evidence does not support its direct activation of estrogen receptors (50). This characteristic provides a unique theoretical perspective for the potential application of peppermint oil in hormone-sensitive disorders—it may avoid certain risks associated with conventional HRT.

In summary, the pharmacological actions of peppermint oil are multi-target and multi-level, encompassing a complex network ranging from molecules (inflammatory cytokines, ion channels) to cells (smooth muscle cells, neurons) and systems (HPO axis, HPA axis). This provides a robust theoretical foundation for its exploratory applications in female reproductive health, particularly in the management of dysmenorrhea and menopausal symptom relief. Of particular note, current evidence suggests that the effects of peppermint oil may be independent of the classical estrogen receptor pathway. If validated in future studies, this hypothesis could offer new perspectives for evaluating its suitability in hormone−sensitive populations.

## 3 Clinical application evidence

RCTs have provided preliminary clinical evidence for the application of peppermint oil in the management of menstrual disorders and menopausal symptoms, although research progress varies considerably across different indications. Evidence in the field of dysmenorrhea is relatively concentrated, while research on menopausal symptoms (especially vasomotor symptoms) is still in the early exploration stage.

### 3.1 Primary dysmenorrhea: sufficient evidence but methodological heterogeneity

Existing evidence consistently supports the value of peppermint oil in alleviating primary dysmenorrhea. A systematic review that included five RCTs involving a total of 499 participants indicated that mint intervention significantly reduced the severity of dysmenorrhea and improved associated symptoms (p < 0.05) (14). In terms of comparative efficacy, peppermint oil capsules (187 mg) demonstrated comparable effectiveness to the conventional non−steroidal anti−inflammatory drug mefenamic acid in relieving dysmenorrhea (51), suggesting that peppermint oil may serve as an adjunct to first−line therapies in the future. However, current evidence is insufficient to support its use as an independent alternative to conventional treatment. Furthermore, observational studies have found that regular premenstrual use of ginger oil reduces dysmenorrhea scores (52). Ginger oil is a botanical therapy belonging to the same category as peppermint. This result provides a reference for the potential value of plant essential oils in the management of dysmenorrhea, but it should be noted that its composition is different from that of peppermint oil, and therefore the results cannot be directly extrapolated.

The diversity of administration routes is an important feature of the clinical application of peppermint oil. Existing studies have involved three primary routes: inhalation, topical massage, and oral capsules. **Table 1** summarizes the specific intervention protocols and implementation details of peppermint oil used in relevant clinical studies. Preliminary studies have shown that both single essential oils (e.g., grapefruit essential oil (7), peppermint essential oil (14)) and blended essential oils (e.g., peppermint-containing mixtures (25)), administered via inhalation or abdominal massage, have been associated with reduced dysmenorrhea scores in some trials. Notably, blended essential oils combined with abdominal massage may produce superior analgesic effects compared with single essential oils (14, 25), suggesting synergistic interactions among different essential oil components. However, this finding requires validation in larger samples. It should be noted that some cited studies involve non−peppermint oil components, such as grapefruit essential oil (7), and their evidence serves only as background reference and should not be directly generalized to peppermint oil. Regarding peppermint oil specifically, current research trends are focusing on two directions: first, optimizing oral formulations (e.g., developing oral essential oil capsules to enhance bioavailability (2, 51)), and second, exploring the mechanisms underlying the synergistic effects of blended essential oils (25).

**Table 1:** Summary of key clinical studies on peppermint oil for the relief of primary dysmenorrhea.

Author (year)	Study type	Sample size	Intervention (dosage/concentration/frequency/duration)	Control group	Main outcomes	Limitations	References
Lagziwa et al. (2025)	Systematic review	5 RCTs involving a total of 499 participants	Massage/oral administration, with forms and dosages varying across original studies.	Placebo/Routine treatment	1. Significantly reduced pain severity and improved associated symptoms (p < 0.05).	1. High heterogeneity among included studies.	(14)
Heshmati et al. (2016)	Double-blind clinical trial	90 female college students (aged 18–25 years)	Peppermint capsules, 330 mg three times daily from day 1 to day 3 of the menstrual cycle	Placebo	1. Pain severity significantly reduced.	1. The inclusion of only Iranian female college students limits the generalizability of the findings to broader populations.	(69)
Masoumi et al. (2016)	Randomized double-blind crossover trial	127 female students with a history of primary dysmenorrhea	Peppermint capsules (187 mg/capsule), three capsules taken once daily for 3 consecutive days starting from the onset of menstruation	Mefenamic acid (250 mg every 8 hours, three times daily)	1. Efficacy comparable to control group.	1. The details of blinding implementation are not well documented.	(51)
Song et al. (2018)	Systematic review/Meta-analysis	21 studies	Peppermint−containing blended essential oils administered via abdominal massage or inhalation. For massage, the protocol started one week before menstruation or on days 1−2 of menstruation, once daily for 10−15 minutes per session. For inhalation, the protocol involved daily inhalation for 5−30 minutes during the menstrual period.	Placebo/Routine treatment	1. Compound oil combined with massage showed optimal analgesic effect.	1. Non-uniform intervention protocols across original studies.	(25)
Sultan et al. (2021)	Randomized controlled trial	90 adolescent females aged 13–22 years	Peppermint capsules (250 mg/capsule), three capsules daily for 5 consecutive days, starting 2 days before the expected onset of menstruation.	Placebo capsules and ginger capsules	1. Pain severity was reduced after the intervention compared with baseline and with the placebo control group, with a greater reduction observed in the ginger group.	1. The inclusion of only Pakistani adolescent females limits the generalizability of the findings to broader populations, and the specific methods of blinding and randomization were not reported.	(70)
Negara et al. (2021)	Cross-experimental study	64 high school students	Ginger oil (a phytotherapy belonging to the same category as peppermint), 5 mL per application, applied topically at bedtime, from 5 days before menstruation until day 2 of the menstrual period.	—	1. Dysmenorrhea pain scores were significantly reduced (p = 0.0001).	1. Non-peppermint oil direct study, only indirectly suggested the potential for cycle regulation.	(52)

However, existing studies still have notable limitations in sample size, blinding implementation, and standardization of intervention protocols. There is considerable variation across studies in essential oil types, administration concentrations, treatment duration, and outcome measures (15, 53–55), which makes direct comparison of results difficult and limits the conduct of high−quality meta−analyses. Furthermore, evidence directly targeting the regulatory effects of peppermint oil on the menstrual cycle (e.g., cycle regularity, menstrual blood loss) remains scarce. These methodological heterogeneities and evidence gaps represent major barriers to the clinical translation of peppermint oil for dysmenorrhea, highlighting the urgent need for future targeted investigations.

### 3.2 Menopausal symptoms: local evidence and systemic gaps

The application of peppermint oil in the management of menopausal symptoms remains in the early exploratory stage, with existing evidence exhibiting a marked imbalance across indications. Current evidence primarily supports its ameliorative effects on certain genitourinary symptoms. A randomized controlled trial of 63 perimenopausal and postmenopausal women found that peppermint oil massage (1.5% concentration diluted in sweet almond oil, administered as hand and arm massage twice weekly for 30 minutes per session over 4 weeks) significantly improved genitourinary symptoms (e.g., vaginal dryness, urinary frequency) compared with lemon essential oil (p=0.001) (15). This provides preliminary evidence for the application of peppermint oil in alleviating local menopausal symptoms. However, clinical evidence for core vasomotor symptoms (e.g., hot flashes, night sweats) is completely lacking; existing hypotheses are based solely on anti-inflammatory propertie (56–58) and have not yet received any clinical validation. In addition, the smooth muscle relaxant effect of peppermint oil may also contribute to alleviating common gastrointestinal disturbances associated with menopause (59). Systematic reviews have confirmed that peppermint oil can relieve functional gastrointestinal disorders through its smooth muscle relaxant properties (60). Studies have shown that peppermint oil significantly improves abdominal pain and symptoms related to intestinal spasms in patients with diarrhea−predominant irritable bowel syndrome (61, 62). Although these findings originate from populations with functional gastrointestinal diseases, the spasmolytic mechanism of peppermint oil on intestinal smooth muscle shares mechanistic commonalities. Its potential value for menopausal−related gastrointestinal disturbances remains to be investigated. Furthermore, safety profiles differ across *Mentha* species (63). For instance, *Mentha pulegium* carries significant hepatotoxicity due to its high pulegone content, which is distinct from the safety profile of *Mentha* × *piperita* (64).

Regarding the management of menopausal psychological symptoms, indirect evidence suggests that peppermint oil may influence anxiety-related behaviors through the GABA pathway (48, 49), providing a theoretical possibility for its intervention in menopausal mood disorders. However, direct clinical evidence in this regard remains scarce. Future studies may incorporate techniques such as electroencephalography and neurobiochemical assays to further explore its regulatory effects on central neurotransmitters and stress hormones, including serotonin and cortisol (65, 66). Furthermore, whether the effects of peppermint oil on menopausal symptoms depend on estrogen receptor pathways remains inconclusive. Current evidence suggests that it does not directly activate estrogen receptor α or β (50); however, this observation is derived from limited *in vitro* studies and is insufficient to confirm its safety in a clinical context, particularly for hormone−sensitive populations. Therefore, dedicated safety studies targeting this specific population are urgently needed.

In clinical practice, the combination of peppermint oil with conventional medications has shown synergistic potential. Its antispasmodic mechanism (TRPM8 activation) theoretically supports combination therapy with conventional antispasmodics (51, 67); however, the effects of different essential oil combinations vary (7, 25), necessitating individualized design protocols and rigorous drug interaction studies. Furthermore, regarding optimization of blended essential oils, one study demonstrated that peppermint oil combined with lemongrass essential oil at a specific ratio (70:30) exhibited synergistic effects in experimental settings (68). However, this finding is preliminary and requires further validation in humans to determine its clinical significance. It should be noted that the non−peppermint oil studies cited here (e.g., lemon and lemongrass essential oils) serve only as background for compound formulation or control design, and their results are not directly generalizable to peppermint oil.

## 4 Research bottlenecks and challenges: four major obstacles in the translation from empirical medicine to evidence-based practice

Although peppermint oil demonstrates substantial potential in the management of dysmenorrhea and menopausal symptoms, its clinical application still faces numerous controversies and challenges. These challenges can be categorized into four core bottlenecks: lack of safety evidence, insufficient standardization and bioavailability, fragmented clinical evidence, and limited mechanistic understanding. These bottlenecks are interrelated and mutually reinforcing, collectively constituting major obstacles in the transition from empirical medicine to evidence-based practice. **Table 2** summarizes the main challenges and future directions of current research.

**Table 2:** Summary of major challenges and future directions in current research.

Challenge category	Specific issues	Future directions	References
Safety in special populations	1. Unclear risk of use in patients with hormone-sensitive tumors; 2. Lack of safety evidence for pregnancy/preconception	Conduct clinical trials in breast cancer survivors, endometriosis, and pregnant women	(71, 72)
Long-term safety	1. Impact of long-term high-dose intake on liver/kidney function, nervous system, and metabolic homeostasis not evaluated	Large-sample, long-term observational studies and registry research	(73)
Standardized production	1. Inconsistent composition due to different extraction processes; 2. Lack of fingerprint quality control standards	Establish industry standards based on fingerprint profiles and marker component content	(32, 75–77)
Bioavailability	1. Rapid metabolism and low absorption of menthol	Develop novel delivery systems such as liposomes and polymer nanoparticles	(79)
Clinical evidence	1. Small sample sizes, non-uniform intervention protocols, heterogeneous outcome measures	Conduct international multicenter, large-sample placebo-controlled RCTs; establish core outcome set (COS)	(15, 53–55)
Mechanism research	1. Fragmented multi-target hypothesis; 2. Unclear impact on hormonal pathways	Integrate multi-omics technologies including genomics, metabolomics, and microbiomics	(42, 50, 66)

### 4.1 Safety controversies in special populations

The safety controversies surrounding peppermint oil first manifest in the uncertainty regarding its dose-effect relationship. Although its primary active component, menthol, has demonstrated potential anticancer activity against various solid tumor cell lines *in vitro* studies, significant controversy remains regarding its dose safety (71, 72). More critically, existing studies have largely focused on short-term efficacy assessments, with a marked lack of evidence on the safety and tolerability of long-term use. Studies have shown that peppermint oil exerts a preventive effect against calcium oxalate kidney stones. However, the potential impacts of long-term, high-dose menthol intake on liver and kidney function, the nervous system, and metabolic homeostasis have not been systematically evaluated (73). Furthermore, differences in long-term toxicity across different administration routes (topical, oral, inhalation) remain unclear, necessitating large-scale, long-term observational studies to address this gap. The most critical safety gap concerns special populations, including breast cancer survivors, individuals with endometriosis, and women attempting to conceive or who are pregnant. Due to the lack of clear understanding of peppermint oil’s hormonal activity, its use in these populations carries substantial safety uncertainty. Another significant safety controversy involves the minimum appropriate age for using peppermint oil in the adolescent population. Menthol and related compounds have been reported to interact with transient receptor potential channels (35, 36) and influence neuroendocrine pathways (42), suggesting potential endocrine−disrupting effects. However, no dedicated studies have systematically evaluated the safety of peppermint oil in individuals under 18 years of age, nor are there age−specific dosage recommendations. Dysmenorrhea management is common in adolescents, but current evidence is derived almost exclusively from adult populations (14), making extrapolation highly uncertain, particularly with regard to potential interference with the still−maturing HPO axis. A randomized controlled trial involving adolescents aged 13−22 years with primary dysmenorrhea showed that peppermint capsules (three capsules daily for 5 consecutive days during menstruation) reduced dysmenorrhea scores, though the effect was inferior to ginger, and no serious adverse events were reported (70). Moreover, the study had limitations including a limited sample size, short intervention duration, lack of hormonal level measurements, and no assessment of menstrual cycle regularity, making it insufficient to confirm the long−term safety of peppermint in adolescents; further validation is needed. Furthermore, the potential impact of peppermint oil on fertility remains unclear, with no relevant human studies available and limited animal evidence yielding inconsistent results. For instance, an aqueous extract of *Mentha rotundifolia* significantly reduced sperm count, motility, and viability in rats (74), while another study reported that peppermint oil exposure might affect follicular development in rats (41). Although these findings suggest that *Mentha* species may exert certain effects on reproductive function, the available evidence remains very limited. Moreover, the *Mentha* varieties, extraction methods, and experimental conditions vary across studies, making direct comparisons of the results difficult. To date, no studies have evaluated the effects of peppermint oil on ovarian reserve, embryo development, or conception rates. Future research must prioritize rigorous clinical trials targeting these special populations to establish the specific risk-benefit profile of its application.

### 4.2 Dual bottlenecks in clinical translation: standardization and bioavailability

Standardized production represents one of the key technical challenges constraining the clinical translation of peppermint oil. The chemical composition and biological activity of peppermint oil are influenced by multiple factors, including variety, geographical origin, and extraction technique. **Table 3** summarizes the effects of different extraction techniques and varieties on the chemical composition and biological activity of peppermint oil. Studies have shown that essential oils obtained by conventional water distillation versus modern techniques (e.g., ultrasound-assisted extraction, nanoemulsion preparation) exhibit differences in the content of major components (e.g., menthol, menthone) and in bioavailability (32, 75–77). For example, peppermint oil extracted by water distillation contains 41.6% menthol (76), whereas electroporation pretreatment combined with solvent extraction can substantially enhance the extraction yield of hydrophobic components (78). Furthermore, the yields of different extraction methods (e.g., Soxhlet extraction, ultrasound-assisted extraction) are influenced by solvent polarity (77). Due to the lack of standardized production and quality control systems, it is difficult to ensure consistency in the stability of key active components and clinical efficacy of peppermint oil products from different sources and preparation methods. This directly affects research reproducibility and therapeutic reliability.

**Table 3:** Effects of different extraction technologies/varieties on the chemical composition and bioactivity of peppermint oil.

Category	Specific method/variety	Key composition changes	Impact on bioactivity/yield	References
Extraction technology	Novel biomolecular hydrodistillation (BMHD) vs. traditional hydrodistillation	Menthol content increased by 10%, oil yield increased by 130%	Yield improved without affecting physical parameters	(32)
Extraction technology	Hydrodistillation	Menthol 41.6%, menthone 24.7%	Conventional extraction, stable composition	(76)
Extraction technology	Electroporation pretreatment + solvent extraction	Extraction rate of hydrophobic components increased by 77%	Extraction efficiency improved	(78)
Extraction technology	Soxhlet extraction, ultrasound-assisted extraction, cold maceration	Yield influenced by solvent polarity (70% ethanol, ethyl acetate, water)	Different solvents affect phenolic compound yield	(77)
Mint variety	Peppermint, spearmint, cornmint	Potential significant differences in chemical composition	Directly affects bioactivity	(29–31)
GRAS certification	Peppermint oil (FEMA 2848), spearmint oil (FEMA 3032), cornmint oil (FEMA 4219)	—	Generally Recognized as Safe	(30)

In addition, components such as menthol suffer from low bioavailability and rapid metabolism, further limiting clinical translation. Studies have shown that nanocarrier technologies (e.g., liposomes, polymeric nanoparticles) can enhance their ability to penetrate biological barriers, thereby enabling targeted delivery (79); while controlled-release systems (e.g., sustained-release microspheres) help maintain stable plasma drug concentrations and prolong the duration of action (80, 81). Furthermore, the compounding ratio of essential oils (e.g., mixing peppermint oil with lemongrass oil at a 70:30 ratio) may significantly alter efficacy (68). Therefore, establishing standardized production processes and quality evaluation systems is essential. Together with the development of efficient and controllable novel drug delivery systems, this represents the pathway to overcoming the clinical translation bottlenecks of peppermint oil and achieving stable and reliable clinical efficacy.

### 4.3 Fragmented clinical evidence and methodological heterogeneity

Current studies also exhibit significant methodological heterogeneity, which severely hampers evidence synthesis and evaluation. Specific manifestations include: inconsistent intervention protocols (variety, dose, concentration, duration), diverse administration routes (massage, inhalation, oral), and inconsistent outcome measures (pain scales, quality of life questionnaires, follow-up time points) (15, 53–55). This heterogeneity makes direct comparisons of study results difficult and precludes the conduct of meta-analyses, thereby reducing the strength of the available evidence. Future research urgently requires the establishment of a unified core outcome set, including standardized symptom assessment scales, administration protocols, and follow-up measures, to improve evidence quality and comparability.

### 4.4 Limitations in mechanistic understanding and unclear core targets

Despite the existence of multiple hypotheses, a comprehensive understanding of the mechanisms underlying the effects of peppermint oil on menstrual disorders and menopausal symptoms remains insufficient. Existing studies have suggested the potential involvement of multiple pathways, including prostaglandin synthesis, modulation of uterine smooth muscle calcium channels, TRP channel activation, and neuroendocrine regulation (35–37, 42). However, these findings are largely fragmented and derived from isolated *in vitro* or animal experiments, lacking systematic validation at the whole-animal level or in humans. A key question remains unresolved: whether and how peppermint oil affects the hormonal network (e.g., estrogen and progesterone pathways). The answer to this question directly determines the potential risks and benefit boundaries of its application in hormone-sensitive disorders. Available evidence suggests that its effects may be mediated through estrogen-independent pathways (50), but clinical safety data in these populations are lacking. This knowledge gap and the aforementioned lack of safety evidence in special populations are mutually constraining: the unclear mechanisms preclude prediction of risks in special populations, while the absence of clinical data in turn limits the targeting of mechanistic studies.

## 5 Comparison with conventional treatments

The above mechanistic limitations make it difficult to establish the clinical positioning of peppermint oil at present. To clarify its potential role in clinical practice, a brief comparison with conventional treatment options in terms of efficacy and safety is of important reference value. **Table 4** summarizes the application of peppermint oil versus conventional treatment regimens in the management of primary dysmenorrhea and menopausal symptoms.

**Table 4:** Comparison of peppermint oil with conventional treatment regimens in the management of primary dysmenorrhea and menopausal symptoms in women.

Comparison dimension	Indications	Conventional treatments	Peppermint Oil
Efficacy evidence	Primary dysmenorrhea	1. NSAIDs: valdecoxib (20/40 mg) was superior to placebo and comparable to naproxen sodium 550 mg (82). 2. Oral contraceptives: 47.2% of patients achieved a pain−free state after one month of treatment (83).	1. A small−sample trial showed comparable efficacy to NSAIDs (51), but the strength of evidence is clearly insufficient. 2. A systematic review supported a reduction in pain scores (p < 0.05) (14).
	Menopausal symptoms	1. HRT: reduced hot flush frequency by approximately 77%, significantly outperforming placebo (85, 86). 2. Non−hormonal agents (SSRIs, gabapentin): have moderate efficacy (88).	1. One trial has shown efficacy for genitourinary symptoms (15). 2. No clinical evidence supports efficacy for core vasomotor symptoms (hot flashes/night sweats).
Safety/Adverse Reactions	Primary dysmenorrhea	1. NSAIDs: gastrointestinal adverse effects occur in 10–30% of users; long−term use increases cardiovascular risk (11, 12). 2. Oral contraceptives: contraindications include thromboembolic events and certain cancers (84).	1. Non−hormonal mechanism of action (48, 50); short−term tolerability appears favorable, but long−term safety data are lacking.
	Menopausal symptoms	1. HRT: long-term use increases the risks of breast cancer, cardiovascular events, and stroke (87). 2. Non-hormonal agents: side effects include nausea and dizziness (88).	1. No evidence currently indicates direct activation of estrogen receptors (48, 50); this provides a theoretical avenue for exploration in hormone−sensitive patients, but clinical safety data are lacking.
Clinical positioning	–	1. First−line treatment with well−established efficacy, but limited by adverse effects and restrictions on applicable populations.	1. Currently only suitable as an adjunctive therapy for patients who are intolerant to or have contraindications to conventional treatment; not a substitute for standard care.

In the management of dysmenorrhea, NSAIDs and oral contraceptives are currently the first-line treatments in clinical practice (10), both with well-established efficacy. A crossover trial demonstrated that valdecoxib (20 mg and 40 mg) was superior to placebo in relieving primary dysmenorrhea and comparable in efficacy to naproxen sodium 550 mg (82). RCTs have also confirmed that 47.2% of patients with dysmenorrhea achieved a pain-free state after one month of oral contraceptive therapy (83). However, NSAIDs are associated with gastrointestinal adverse effects in 10–30% of users, and long-term use increases cardiovascular risk (11, 12). Oral contraceptives carry contraindications including thromboembolic events and certain cancers (84). In contrast, the evidence supporting comparable efficacy of peppermint oil to NSAIDs in relieving dysmenorrhea is derived from only a small-scale trial (51), with clearly insufficient strength. Nonetheless, its non-hormonal mechanism offers a potential option for patients who cannot tolerate conventional therapies.

For menopausal symptom management, a Cochrane systematic review showed that HRT reduces hot flush frequency by approximately 77%, significantly outperforming placebo (85, 86). However, long-term use increases the risks of breast cancer, cardiovascular events, and stroke (87). Non-hormonal agents, such as selective serotonin reuptake inhibitors (SSRIs) and gabapentin, have moderate efficacy but are limited by side effects including nausea and dizziness (88). For peppermint oil, one trial has shown benefit for genitourinary symptoms (15), with no clinical evidence supporting its efficacy for core vasomotor symptoms.

From the perspective of patient preference, those who favor non-pharmacological interventions or wish to reduce their reliance on conventional medications appear to be more receptive to complementary therapies, including peppermint oil. However, patient expectations should be grounded in available evidence, which currently cannot ensure consistent therapeutic outcomes. Therefore, based on current data, peppermint oil is only suitable as an adjunctive therapy for patients who are intolerant to or have contraindications to conventional treatments. Well-designed head-to-head comparative trials are needed in the future to clarify its relative efficacy and safety profile.

## 6 Future perspectives

Based on the aforementioned research challenges, future studies should focus on the following key directions to advance the evidence base and standardize the clinical application of peppermint oil.

### 6.1 Conducting high-quality, standardized clinical studies

To address the fragmented clinical evidence and methodological heterogeneity discussed above, there is an urgent need for international multicenter, large−sample, placebo−controlled RCTs to strengthen the evidence base for the efficacy of peppermint oil. Study designs should prioritize specific populations (e.g., severe dysmenorrhea, specific menopausal symptom subtypes) to clearly define the boundaries of efficacy. Establishing a core outcome set (COS) is critical for evidence synthesis, and should include standardized symptom assessment scales, intervention protocols (essential oil variety, dose, route, duration), and follow-up measures to improve comparability across different studies. Furthermore, RCTs specifically designed with core menopausal symptoms, such as hot flashes and night sweats, as primary outcome measures are urgently needed to fill the current research gap.

### 6.2 Exploratory directions: systematic elucidation of molecular mechanisms using multi−omics technologies

As an exploratory hypothesis, future research may attempt to employ multi−omics technologies for systematic investigation, in order to provide new entry points for overcoming the current cognitive limitations in mechanistic understanding. Specifically, the following approaches are recommended: (1) integrating genomics and epigenomics to analyze the effects of peppermint oil on the expression of HPO axis-related genes (42); (2) employing metabolomics to quantify its modulation of hormone metabolites, inflammatory cytokines, and stress hormones (55, 66); (3) incorporating microbiomics to investigate its regulatory effects on the vaginal/gut microbiota and the association with symptom relief (89). In addition, electroencephalography or functional magnetic resonance imaging could be employed to explore the immediate effects of inhaled peppermint oil on emotion−regulating brain regions (e.g., amygdala, prefrontal cortex) (65, 66). If implemented in future studies, the multi−omics integration strategy outlined above may help systematically elucidate the multi−target network of peppermint oil, particularly providing clues regarding its interactions with sex hormone synthesis and metabolic pathways, informing the safety boundaries for its clinical application.

### 6.3 Advancing standardized production and the development of novel drug delivery systems

Establishing industry standards based on fingerprint profiles and marker component content is an important foundation for promoting the standardized clinical application of peppermint oil. Quality control specifications for peppermint oil products should be developed, clearly defining the botanical source (e.g., *Mentha* × *piperita* vs. *Mentha spicata*), extraction technique, and content ranges and ratios of key components (menthol, menthone). This would ensure the stability of key active ingredients across products from different sources and batches, thereby improving the reproducibility of research findings. Simultaneously, interdisciplinary research at the interface of pharmaceutics and pharmacology should be encouraged to develop nanotechnology-based novel drug delivery systems. These include: (1) nanolipid carriers, to enhance transdermal absorption rates and uterine-targeted concentrations; (2) oral sustained-release microspheres, to maintain stable plasma drug concentrations and reduce gastrointestinal irritation; and (3) vaginal/rectal suppositories, for local administration targeting genitourinary syndrome of menopause. The development of these novel formulations is a key approach to improve the bioavailability of peppermint oil and achieve targeted delivery and controlled release. This may offer new options for managing symptoms such as dysmenorrhea and hot flashes.

### 6.4 Improving long-term safety and evidence in special populations

Future research should conduct large-sample, long-term observational studies and registry studies to systematically evaluate the hepatic, renal, and neurological toxicities as well as metabolic effects associated with long-term use of peppermint oil. Differences in long-term safety across different administration routes also require clarification. Of particular importance, clinical trials should be prioritized in the following special populations: (1) breast cancer survivors, to assess potential interference with estrogen pathways and interactions with endocrine therapies; (2) individuals with endometriosis, to evaluate effects on ectopic lesions; (3) women attempting to conceive or who are pregnant, to assess reproductive toxicity and embryo safety; and (4) adolescents with dysmenorrhea, to define the safety window for long-term use. These studies will contribute to clarifying the contraindications and risk-benefit ratio of peppermint oil, which may serve as a reference for individualized decision-making in clinical practice.

## 7 Conclusions

Peppermint oil, with its multi-target pharmacological properties, shows noteworthy research promise in the relief of primary dysmenorrhea and menopausal genitourinary symptoms. Its anti-inflammatory, antioxidant, and smooth muscle relaxant effects are achieved through multiple mechanisms, including modulation of calcium channels, inhibition of inflammatory pathways, and regulation of the neuroendocrine network. However, current research remains in the early stages of transitioning from empirical use to evidence-based evaluation. It faces key bottlenecks, including fragmented clinical evidence, insufficient mechanistic understanding, lack of product standardization, and scarcity of long-term safety data. The core task of future research is not simply to repeatedly validate its efficacy, but to systematically answer three fundamental questions: how does it work, who will benefit from it, and how can it be applied safely and effectively? Gradual elucidation of these issues will help promote the standardized application of peppermint oil in managing dysmenorrhea and menopausal symptoms. Achieving this goal will require deep synergy between basic and clinical research, as well as interdisciplinary integration across pharmacy, chemistry, and medicine.

## Author's note

To ensure accurate cultural representation, the authors' names are presented in Pinyin followed by their original Chinese characters: Linfeng Mo (莫林烽), Shuya Ye (叶舒雅), Yan Qin (覃艳), Huihuang Shen (沈慧煌), Huiyi He (何惠仪), Wenyan Hong (洪文艳), and Hongmei Wu (吴红梅).
